# A Case of Myelin Oligodendrocyte Glycoprotein Antibody-Associated Optic Neuritis Responsive to Intravenous Immunoglobulin (IVIG) Therapy in a Pediatric Patient

**DOI:** 10.7759/cureus.43218

**Published:** 2023-08-09

**Authors:** Rochita Kadam, Waseem Fathalla, Syed A Hosain, Reem Al BinAli

**Affiliations:** 1 Pediatrics, Sheikh Shakhbout Medical City, Abu Dhabi, ARE; 2 Pediatric Neurology, Sheikh Shakhbout Medical City, Abu Dhabi, ARE; 3 Pediatric Neurology, Alberta Children's Hospital, Alberta, CAN

**Keywords:** pediatric, chronic relapsing inflammatory optic neuropathy(crion), intravenous immunoglobulin therapy, myelin oligodendrocyte glycoprotein-associated disorders, myelin oligodendrocyte glycoprotein antibody, optic neuritis

## Abstract

We present a case of an eight-year-old boy who presented with complaints of headache, blurry vision, and eye pain. Ophthalmological exams and magnetic resonance imaging confirmed the presence of optic neuritis. Initial cerebrospinal fluid analysis was negative for all antibodies (Abs) associated with optic neuritis and other acute demyelinating syndromes, including anti-myelin oligodendrocyte glycoprotein Ab (anti-MOG-Ab). The child was treated with a course of pulse methylprednisolone therapy for five days, with significant improvement in his symptoms. However, the child went on to have a recurrent episode of optic neuritis one month after his initial presentation. Hence, investigations targeting immunological biomarkers were repeated and turned out to be positive for anti-MOG-Abs with elevated titers. The child was diagnosed with MOG-Ab-associated optic neuritis presenting as chronic relapsing inflammatory optic neuropathy (CRION). He was then started on maintenance intravenous immunoglobulin (IVIG) therapy as a disease-modifying therapy, following which he has not had any further relapses over two years.

## Introduction

Optic neuritis is one of the most common forms of acquired demyelinating syndromes in childhood. It presents as unilateral or bilateral vision changes, often associated with pain. It usually points toward an underlying demyelinating syndrome [1]. Chronic relapsing inflammatory optic neuropathy (CRION) is characterized by recurrent episodes of optic neuritis, accompanied by clinical symptoms and corresponding neuroimaging findings. Optic neuritis with myelin oligodendrocyte glycoprotein antibody (MOG-Ab) seropositivity may present with neurological or systemic symptoms. Optic neuritis with MOG-Ab seropositivity may occur with neurological or systemic symptoms and often follows a recurring pattern; therefore, it is classified as CRION [2]. Although the full spectrum of phenotypes and best treatment options are still being determined, it is important to consider MOG-Ab-associated disorders in the differential diagnosis of CRION and seek expert treatment advice when possible.

## Case presentation

**Figure 1:**
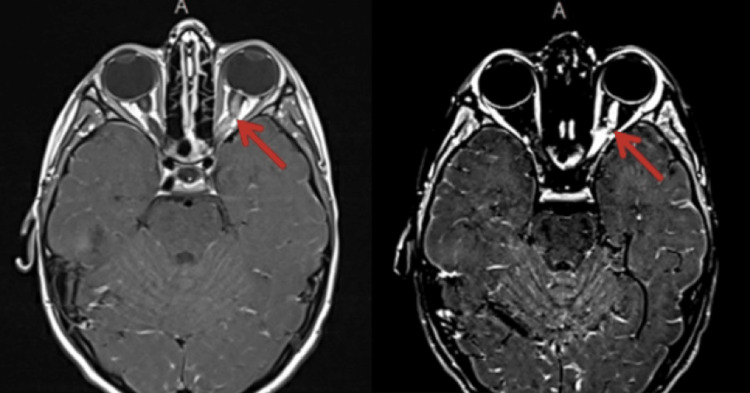
MRI of the brain showing left optic nerve enhancement during the first episode; MOG serology was negative at this time. MOG, myelin oligodendrocyte glycoprotein

An eight-year-old boy, presented with complaints of headaches for two weeks and blurry vision in the left eye for two days. There was associated pain when moving the left eye. The patient had a history of well-controlled focal epilepsy managed with levetiracetam while being otherwise in good health. The patient’s physical examination was remarkable for pain elicited when moving the left eye and for papilledema of the left optic disc. MRI of the brain and orbit revealed an edematous left optic nerve displaying pronounced enhancement, indicative of optic neuritis. There was also mild enhancement of the central part of the right optic nerve (Figure 1). Pattern visual evoked potentials (PVEPs) showed an abnormal cortical response during left eye stimulation. A lumbar puncture showed opening pressure of 31 cm H_2_O, which was considered high for a child of this age. The cerebrospinal fluid analysis revealed a WBC count of 2 × 10^6 ^L^-1^, a protein level of 0.12 g/dL, and an IgG level of 65 mg/dL, all within the normal range for his age. The immunology panel, including anti-Aquaporin-Ab and anti-MOG-Ab, was eventually negative.

The child was started on a high dose of 30 mg/kg of methylprednisolone for five days, with gradual tapering over two weeks. The child reported significant improvement in vision following the initiation of steroid therapy.

**Figure 2:**
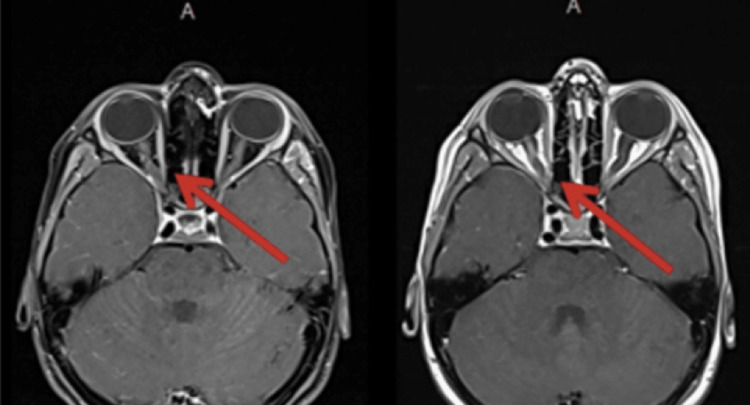
MRI of the brain showing the right optic nerve enhancement during a recurrent episode; MOG serology was positive at this time. MOG, myelin oligodendrocyte glycoprotein

After one month of the initial presentation, the child returned with similar complaints of eye pain and blurry vision, suggesting a relapse. An MRI of the brain and orbit confirmed the presence of right optic neuritis, exhibiting enhancement of the right optic nerve (Figure 2). In addition, his PVEP displayed an abnormal cortical response on the right, along with a recovery of latency responses in the left optic pathway.

He was treated with methylprednisolone pulse therapy of 30 mg/kg for five days once more, resulting in a complete recovery of his symptoms. His anti-MOG-Ab results returned positive with a titer of 1:100.Subsequently, the decision was made to initiate monthly intravenous immunoglobulin (IVIG) infusion therapy as a disease-modifying intervention, in accordance with the consensus of the E.U. Pediatric MOG Consortium [3]. The child tolerated and responded well to the treatment. After one year, a repeat of his anti-MOG-Ab titer was still positive with a titer of 1:100. Due to the absence of clinical relapses, IVIG therapy was gradually spaced out over the second year. The child continues to receive regular quarterly IVIG infusions. Thus far, there have been no relapses over two years. His latest anti-MOG-Ab titer stands at 1:40 (Table 1).

**Table 1:** Interval decrease in anti-MOG-Ab titers after commencing IVIG therapy. Ab, antibody; MOG-Ab, myelin oligodendrocyte glycoprotein antibody; IVIG, intravenous immunoglobulin

Time of presentation	MOG serology	Ab titers
First episode of optic neuritis	Negative	<1:20
Second episode of optic neuritis	Positive	1:100
After two years of IVIG maintenance therapy	Positive	1:40

## Discussion

In the pediatric population, the incidence of optic neuritis is approximately 0.15-0.57 per 100,000 children annually. It tends to be more prevalent among adolescents than younger children. Additionally, studies have demonstrated a female predominance, similar to that seen in the adult population [1].

In recent times, there is growing interest and research directed toward optic neuritis. This has partly been due to the expanding clinical spectrum of the anti-MOG-Ab-associated disease in children. MOG is a transmembrane protein in the myelin sheath of the central nervous system. It has various functions, including the regulation of oligodendrocyte microtubule stability, myelin adhesion, and the activation of the complement cascade [1].

Anti-MOG-Ab is associated with a wide spectrum of clinical presentations. It is linked to both monophasic and relapsing forms of acute disseminated encephalomyelitis (ADEM), transverse myelitis, and optic neuritis. When the biomarker was initially found, it was most often associated with ADEM in children. However, recent studies have shown that Ab is more closely associated with optic neuritis [1]. Clinically and radiologically, MOG-Ab-associated optic neuritis tends to exhibit greater involvement of the anterior optic pathways, while showing a tendency to spare the optic chiasm and optic tracts, as demonstrated in our case.

On examination, optic disc edema is more prevalent in MOG-Ab-associated optic neuritis in comparison to other demyelinating Abs. It is usually present in up to 85% of cases [1]. In a retrospective study by Giacomini et al., it was seen that the presence of optic disc swelling at baseline presentation was strongly associated with anti-MOG-Ab seropositivity. Moreover, children with optic neuritis who were seropositive for anti-MOG-Abs demonstrated a better recovery compared to the seronegative ones [4]. Thus, in our opinion, it could be essential to assess for anti-MOG-Abs in a child presenting with acute vision changes, indicating optic neuritis, so that a prompt diagnosis can be made and treatment can be initiated.

Seropositivity of Ab often correlates to the nature of the disease itself. Specifically, persistently raised anti-MOG-Ab titers point toward a more relapsing course than a monophasic one. Recent studies have shown patients with declining anti-MOG-IgG titers, particularly those with seroconversion to anti-MOG-IgG-negative within one to two years, have a significantly reduced risk for relapse [5]. A study conducted in the Netherlands was able to demonstrate that more than 50% of pediatric patients with a relapsing course of the disease remain seropositive for a longer time, as opposed to those with a monophasic course [6]. At present, there are no reliable factors to predict the course of the disease itself based on the presence of the anti-MOG-Ab alone. There is a growing need for studies to find the temporal relationship between seroconversion and disease course. As outlined in our case, the investigations conducted during the first presentation did not show the presence of anti-MOG-Abs, but a repeat analysis performed during a relapse demonstrated seroconversion and detected anti-MOG-Abs. Moreover, the repeat analysis showed a gradual decrease in the titers of Abs, which could correlate to the nonrelapsing course in our case.

The current treatment modalities for anti-MOG-Ab-positive optic neuritis are aimed toward treating acute presentation and reducing the chances of relapses. The standard course of treatment in acute presentations is steroid therapy. In general, anti-MOG-Ab-positive children remain on steroid therapy for four to six weeks after the initial presentation. Most cases of relapses occur within one to two months of cessation of the steroid therapy. In these cases, the decision to commence regular immunotherapy is often considered and initiated based on individual patient factors. Most of the recent studies have indicated that relapse rates are significantly reduced with maintenance immunotherapy with agents like Prednisolone, IVIG, Rituximab, or Mycophenolate Mofetil. A recent retrospective study conducted by Chen et al. showed that IVIG maintenance therapy was most effective with a fewer side effects among all the agents in the reduction of the relapse rate per year [7]. This has proven to be true in our case, as no relapses have occurred on IVIG maintenance therapy.

## Conclusions

This case shows that IVIG therapy can be used to treat MOG-Ab-associated optic neuritis in the pediatric population. As optic neuritis is a common presentation of demyelinating disorders in the pediatric population, prompt investigations for underlying etiologies are of great significance. MOG-Ab-related disorders make up a major fraction of these presentations. Moreover, anti-MOG-Ab seroconversion can often be seen later in the disease course of children presenting with recurrent episodes of optic neuritis. As described in our case, only a repeat analysis for anti-MOG-Abs turned out to be positive with high titers. Furthermore, the commencement of regular IVIG therapy showed good therapeutic benefits with significantly reduced Ab titers over two years in our case. Thus, clinicians should be aware of the importance of testing for anti-MOG-Abs in recurrent episodes of demyelinating conditions like optic neuritis when initial workup fails to yield an appropriate diagnosis, as timely initiation of suitable immunotherapy can result in better prognostication and quality of life in such patients.
